# The Importance of Antibiotics in Facial Fracture Treatment—A Systematic Meta-Review

**DOI:** 10.3390/cmtr18040048

**Published:** 2025-11-03

**Authors:** Martin Bengtsson, Aron Naimi-Akbar, Joakim Johansson-Berggren, Sebastian Dybeck-Udd, Mikael Magnusson, Bodil Lund

**Affiliations:** 1 Department of Oral and Maxillofacial Surgery, University Hospital of Skåne, 221 85 Lund, Sweden; joakim.johanssonberggren@skane.se; 2 Department of Clinical Sciences, Faculty of Medicine, Lund University, 222 22 Lund, Sweden; 3 Health Technology Assessment-Odontology (HTA-O), Faculty of Odontology, Malmö University, 205 06 Malmö, Sweden; aron.naimi-akbar@mau.se; 4 Medical Unit of Plastic Surgery and Oral and Maxillofacial Surgery, Department for Oral and Maxillofacial Surgery and Jaw Orthopedics, Karolinska University Hospital, 171 65 Stockholm, Sweden; sebastian.dybeck-udd@regionstockholm.se (S.D.-U.); bodil.lund@ki.se (B.L.); 5 Department of Specialist Dentistry, Oral and Maxillofacial Surgery, Colloseum and Smile AB, 167 51 Stockholm, Sweden; magnusson.mikael@telia.com; 6 Department of Dental Medicine, Karolinska Institute, 141 57 Huddinge, Sweden

**Keywords:** antibiotic prophylaxis E02.319.162.150, E02.319.703.150, maxillofacial injuries C10.900.300.284.500, C26.915.300.425.500, skull fractures C10.900.300.918, C26.404.750, C26.915.300.745, systematic review V02.912.875, V03.850

## Abstract

This meta-review evaluated the possibility of more specified recommendations in antibiotic treatment through a narrowed focus on facial trauma. The aim was to analyze the effect of different regimens of antibiotic in treatment of skeletal trauma to the face. The knowledge mapping was based on existing systematic reviews (SRs) on trials specified in a PICO: Participants (P): Adults and children, diagnosed with fractures to the facial skeleton. Interventions (I): Antibiotic intervention. Comparator (C): Placebo, no antibiotics. Outcomes (O): Postoperative infection, pain, re-operation, other complications, healing deficiencies, (Oral) Health related Quality of Life, removal of osteosynthesis, adverse reactions. The literature search in PubMed, The Cochrane Library, and Web of Science according to PRISMA resulted in 1487 records. A COVIDENCE selection process resulted in 29 articles retrieved and read in full text revealing 10 articles eligible for evaluated according to ROBIS. Three SRs were considered to have low risk of bias and constituted the final evidence evaluation. The meta-review of these SRs did not provide sufficient support for prolonged antibiotic treatment after surgical intervention of midfacial fractures in comparison with antibiotics only the first day postoperatively. No support for antibiotic treatment for conservatively managed fractures alone was found. This review is limited by a relatively low number of included SRs. However, tendencies in outcomes suggests a restricted duration of antibiotics in treatment of facial fractures.

## 1. Introduction

Severe postoperative infections are a risk for full healing ability of wounded tissues. The incidence of surgical site infections after surgical treatment of facial fractures are reported to occur at an average of 12% (range 0% to 30%) [1]. The constitution of opportunistic bacteria depends on the nature and anatomic region of the injury. Other factors of importance may be environmental factors at the location of the causing accident, characteristic of the trauma force, fracture mobility and complexity, wound contamination, and patient’s general health [2].

The use of antibiotics to prevent postoperative infections can be indicated either because of risk factors correlated to the patients’ health status and/or because the surgical procedure in itself poses an increased risk for infection. In the field of oral and maxillofacial surgery (OMS), empiric antibiotics has been suggested in cases such as complex dental implant surgery, immune-deficient patients, orthognathic surgery, surgery of the temporomandibular joint, and facial trauma [3,4,5,6]. Up to now, several publications have presented findings that have resulted in implemented guidelines [7,8,9].

Sensibility to trauma-induced infections depending on the anatomical location is not recently or thoroughly investigated [10]. Studies on other patient groups, e.g., orthognathic surgery, third molar surgery, dental implant surgery, and overall oral and maxillofacial surgery have revealed a higher sensitivity for postoperative infections in the mandible compared to the maxilla [11,12,13,14,15]. In facial trauma including fractures of the jaws, the patient category has mainly been studied as a composite group or in a separate age group, leading to recommendations that aims for protection against several origins of infections and within a selected sample [16,17,18,19,20]. Commonly, these recommendations in cases with treatment of facial trauma have been based on antibiotic coverage of dermal and/or oral bacteria of the upper airway [21]. Furthermore, there are studies that clarify a possible difference in sensibility to postoperative infections depending on the location of the fracture. E.g., it is known that the risk of infection is higher in tooth bearing areas of the lower jaw compared to the upper jaw.

Simultaneously, a restricted use of antibiotics because of a widespread development of antimicrobial resistance (AMR) is globally recommended calling for higher precision in decision-making regarding antibiotic use [1,22,23,24].

Consequently, high-quality comparative studies that clearly point out treatments and patient categories that safely could be treated without the aid of antibiotics are valuable to provide decision support to presumed reluctant clinicians [25,26]. Even more specified recommendations in antibiotic treatment that are based on a narrowed focus on facial trauma in a systematic review (SR) or meta-review may be possible [27].

The aim of this systematic meta-review was to summarize the outcome in previous SRs of different regimens of adjuvant empiric antibiotic in the treatment of facial fractures. The hypothesis was that the outcome from this analysis would facilitate a clearer guidance to the use of antibiotic treatment in facial traumatology.

## 2. Materials and Methods

This study is a mapping of SRs, and the study protocol was, prior to commencing, registered at Prospero URL (https://www.crd.york.ac.uk/PROSPERO, accessed on 14 March 2023). Dnr: CRD42023404870, entitled; What is the effect of adjuvant antibiotics in treatment of fractures to the facial skeleton? The authors confirm compliance with the Preferred Reporting Items for Systematic reviews and Meta Analyses 2020 statement (PRISMA) [28].

### 2.1. Eligibility Criteria

The present research question was defined using the following PICOT [29]:

Participants (P): Patients, adults, and children, diagnosed with fractures to the facial skeleton.

Interventions (I): Any antibiotic intervention.

Comparator (C): Different antibiotic treatment than the intervention, placebo, no antibiotics.

Outcomes (O): Primary outcome: Postoperative infection.

Secondary outcomes: Pain, re-operation, other complications, healing deficiencies, (Oral) Health related Quality of Life, removal of osteo-synthesis, adverse reactions.

Type of studies (T): Systematic reviews.

The knowledge mapping was based on existing SRs, where the included reviews were allowed to include synthesis of observational cohorts and non-randomized and randomized controlled trials (RCT).

### 2.2. Literature Search

The literature search was performed in collaboration with an information specialist at Malmö University library. The following databases were searched: PubMed, The Cochrane Library, and Web of Science. The search was performed 23 June 2025 and the complete search strategy is presented in Table S1.

### 2.3. Selection Process

Prior to onset of the screening process, all researchers were subjected to an educational session held by experts from the Swedish Agency for Health Technology Assessment and Assessment of Social Services. The retrieved publications from the database searches were subjected for an initial inclusion or exclusion of relevant studies based on title and abstract according to predefined inclusion criteria, which were as follows:

Fractures to the facial/visceral skeleton in adult patients included in systematic reviews.

Systematic reviews with sole soft tissue facial injuries, sole pediatric population, not presented in English, and presenting a mix of injuries to body regions other than the facial were excluded.

At least two reviewers screened each title and abstract. The next step of screening was based on full text assessed for eligibility, again by at least two independent reviewers. Any potential disagreement during the screening process was resolved by a consensus discussion between all reviewers. Studies excluded at this stage, and the reason for exclusion was recorded.

### 2.4. Risk of Bias Assessment

All the studies included in the review were subjected to quality assessment using ROBIS [30]. This instrument consists of 21 items/questions grouped within four domains: (1) study eligibility criteria; (2) identification and selection of studies; (3) data collection and study appraisal; (4) synthesis and findings. For each study, the items/questions were graded to any of five different stages: yes, probably, probably not, no, or missing information. Further on, each of the four domains were, based on the previously graded subgrouped items, evaluated for the risk of bias into high, low, or unclear. After considering the items within the domains and the following domain evaluation, an overall risk of bias was determined, and eventual conflict of interest was noted. Two groups, each constituting three reviewers, individually and for all included studies performed the evaluation according to ROBIS structure. At any case of difference in judgement was noted, a consensus was reached between the groups.

### 2.5. Data Extraction

Data were extracted from all studies subjected to quality assessment were study type, number of included studies in the review, type of fractures included, and risk of bias. The studies were grouped according to lack of systematic reproducible literature search (reviews), systematic reproducible literature search and methodology according to PRISMA guidelines (systematic review), and meta-analysis in addition to systematic approach (systematic review and meta-analysis). Web-based software PROSPERO (The Centre for Reviews and Dissemination, University of York, Alcuin College, York YO10 5DD, England) was used for study registration prior to commencing; Covidence (registered in Australia (ACN 600 366 274, ABN 41 600 366 274), 485 Latrobe Street, Melbourne 3000 Australia) was used for improving the production of this review; and ROBIS evaluation form for assessing the risk of bias in included SR publications (SBU—Statens beredning för medicinsk och social utvärdering. The governmental institute for medical and social evaluations. https://www.sbu.se/sv/granskningsmallar/, accessed on 22 October 2025). Only the studies evaluated to have a low risk of bias were eligible for compilation of results and conclusion.

## 3. Results

**Figure 1:**
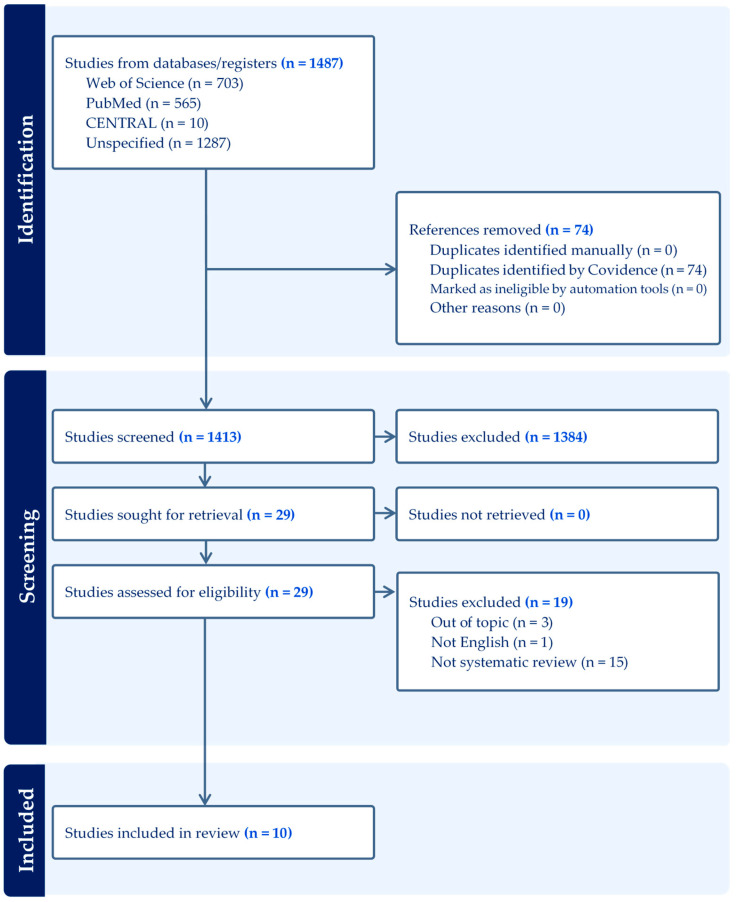
PRISMA 2000 flow chart diagram of the study inclusion process.

The literature search resulted in 1413 records, after removal of 74 duplicates. Out of these 1413 articles, 29 articles meet the inclusion criteria, were retrieved and read in full text (Figure 1).

Ten included studies were evaluated to be eligible for quality assessment using the ROBIS evaluation form (Table 1). Studies excluded from further evaluation with qualitative analysis (*n* = 19) are listed in Table 2.

Out of the ten articles evaluated according to ROBIS, seven were evaluated to have high risk of bias (*n* = 6) or unclear risk of bias (*n* = 1). The conclusions from these were presented but not weighted equally as the other low-risk SRs in suggested clinical implication from the present meta-review. The three SRs considered to have low risk of bias and were subjected to further extraction of outcomes (Table 3).

## 4. Discussion

The present systematic meta-review revealed that three identified SR articles with a low risk of bias did not provide sufficient support for prolonged antibiotic treatment after surgical intervention of facial fractures. In comparison, a prolonged postoperative antibiotic therapy did not provide any advantages compared to only during the first day postoperatively. One of these three SR’s was reporting mainly on mandibular fractures, why it is of less interest regarding evidence based antibiotic regimens in midfacial trauma [32]. However, disregarding this trial did not alter the outcome or the interpretation of the present systematic meta-review.

The three SRs did not provide any support for antibiotic treatment for conservatively managed facial fractures alone. One study presented therapies within OMS and ENT separately and, based on a focus on trauma, was only partly selected to the present meta-review [34]. Accordingly, in the three studies a total number of 6848 individuals were included.

In total, 45 original articles were reviewed in the three SRs that supported the present conclusion. However, reviewing these original studies and excluding duplicates reveal 28 unique publications. Accordingly, the total number of participants included in these 28 studies was 4742. The total sum of subjects is estimated to advocate a strong base for generalization and that a united outcome would be valued as a general recommendation for an antibiotic regimen in treatment of facial fractures.

The conclusion in the SRs presented in Table 3 suggests that the outcome should be interpreted with caution because of limitations in all included studies and that a short course, e.g., maximum 24 h of antibiotic treatment is recommended. Well-constructed studies investigating type and duration of systemic antibiotics are lacking and should be a focus for future scientific efforts.

In addition to facial fractures, surgical treatment in adjacent areas within OMS has previously been evaluated for the need of prophylactic antibiotics. SRs on the role of antibiotics in temporomandibular joint (TMJ) surgery, third molar extractions, implantology, and orthognathic surgery have been presented [11,51,52,53]. In general, these SRs conclude a weak support for the use of empiric antibiotic treatment within the broad field of OMS. Together they present a low risk for postoperative infections and recommend an individual approach in evaluating the patients’ risk. I.e., facial fractures are not one and all the same; open fractures and fractures involving skull base/posterior table of the frontal sinus or ethmoid; or fractures that involve significant contamination or soft tissue involvement may warrant longer duration of treatment.

The present study analyzed the role of empiric antibiotic treatment in combination of surgical treatment of facial fractures. However, it has not focused on differences between subgroups within the area of facial trauma. Few previous studies have analyzed the postoperative sensibility to infections depending on which facial region that was fractured. A retrospective analysis of postoperative infections after orthognathic surgery showed that all recorded postoperative infections were present in the mandible. It was hypothesized that a possible explanation could be due to poorer blood supply and the masticatory forces in the mandible [15]. In a retrospective study on third molar extractions that included over 1000 patients, no postoperative infections were found in the maxilla but a probability of 1.94% in the mandible. A risk factor for mandibular infection was found to be related to the depth of tooth position and might indicate that the amount of surgical invasion and sectioning are related to the increased risk of postoperative mandibular infections [54]. Similar results were presented by Chiapasco et al., who found a postoperative infection rate of 1.5% in the mandible and 0.2% in the maxilla [12]. Furthermore, studies on dental implant treatment have shown a higher postoperative infection rate in the mandible [13,55]. In conclusion, studied cohorts other than facial trauma reveal a higher sensitivity for postoperative infections in the mandible compared to the maxilla. Accordingly, the proposed increased risk of infections in the mandible in combination with the wide scope of the included studies highlights the need for primary studies focusing on antibiotic treatments in other sites of facial traumatology. Such studies may lead to a more effective use of antibiotics.

A possible conflict of interest is that if the clinician has any doubt in putting their patient at risk for infection, which may lead to actions that negatively affect the global spread of AMR [22]. Recently, a follow-up study on adherence to governmental recommendations in a Swedish community showed a positive effect on decreased use of antibiotic treatment in dentistry but stated further potential in health-care recommendations [56].

To date, there is a need for high-quality comparative studies to ensure further development of robust guidelines based on solid scientific evidence [1,32,57].

Mulrow described recommendations for the design of a summary of SR projects, i.e., a meta-analysis in 1994 [58]. An advantage of the meta-analysis is its possibility to increase the power of the analysis by adding several cohorts [45]. However, this also comes with the risk of a divergence of the material that will make any conclusion taken from the meta-analysis doubtful. Thus, it is recommendable to include only primary studies with a similar study protocol in a meta-analysis, which will result in a decreased number of eligible studies. Later on, the recommendations has evolved and the SR approach to knowledge has become more popular. Applying the study design onto different areas of science has also lead to the development of a variety of SRs [59,60].

The method of a meta-review study is relatively novel [61]. The idea of reviewing SRs has emerged from a need for trustworthy, robust guidelines based on solid scientific evidence in health-care decision-making. With an increasing number of SRs, the possibility for an evidence-based approach in therapeutic decisions and in decision-making becomes more complicated [62]. This might result in a larger gap between science, clinicians, and health-care politicians, which in the end will have a negative impact on patient care. Hence, the importance of collecting the present knowledge from a high level of evidence, SRs, and meta-analysis is clear. Besides presenting an overview and summarizing the present evidence, the meta-review also provides a number of independent conclusions from the included SRs, i.e., conclusions on different perspectives like therapeutic efficacy, population and adverse effects [27].

A low number of high-quality SRs, resulting in less geographical coverage, less variation in trauma causality factors, and a lower number of participants represent limitations of the present study. However, with fewer included studies, the number of different study designs also decreases. This might result in a less complicated evaluation of their outcome and lead to a more concise conclusion. However, a meta-review also comes with a risk of performing additional analysis that may lead further away from the recording original studies. Based on this, the present study presents findings without any additional conclusions except what was already stated in the included SRs. Hence, no meta-analysis was performed.

## 5. Conclusions

In conclusion, the reviewed SRs with a low risk of bias did not provide sufficient support for prolonged antibiotic treatment after surgical intervention of facial fractures in comparison with antibiotic only during the first day postoperatively. Neither did the other included SRs. No support for antibiotic treatment for conservatively managed fractures alone was found. Hence, the outcome of the present meta-review reveals that only a restricted duration of empiric antibiotics in treatment of facial fractures are recommended. However, because there are several limiting factors in the studies included, their conclusions as well as the outcome of the present meta-review should be interpreted with caution. Furthermore, it is advisable that a clinical judgment should guide the duration of empiric antibiotic treatment in each case based on patient history, medication, characteristics, injury type, and surgical intervention.

## Figures and Tables

**Table 1:** Characteristics of the studies subjected to qualitative analyses using ROBIS (*n* = 10).

First Author	Year	Type	Included Studies (*n*)	Fracture Location	ROBIS Domain				Overall RoB	Comments	Conclusion
					1	2	3	4			
Blatt [14]	2019	SR	17 of 80	Mandible Maxilla Midface	high	high	high	high	high	In total 80 included studies, whereof 17 regarding trauma.	Guideline is much in need to transfer the indecisive results for antibiotic prophylaxis in dentoalveolar surgery in clinical praxis.
Chaudhry [31]	2021	SR	4	Mandible	low	high	high	high	high	Includes RCT, Observation, retrospective and cohort. No clarified outcome.	No statistical difference in infection rates whether antibiotics are prescribed pre, peri, or postoperatively.
Dawoud [32]	2021	SR/MA	16	Mandible	unclear	low	low	low	low	Includes RCT and retrospective observational. Lack of PICO, age and onset.	No strong evidence for or against prophylactic antibiotic use.
Delaplain [16]	2020	SR/MA	27	Mandible Midface Orbital	low	high	low	high	high	Investigated surgical site infection (SSI) in open reduction and internal fixation of facial fractures (ORIFfx).	No lower rate of SSI associated with PAP for any ORIFfx repair. Postoperative antibiotics for >72 h may increase the SSI risk after mandible fracture repairs.
Goormans [17]	2022	SR	16	Mandible Midface Orbital Frontal	low	low	low	low	low	Investigates systemic antibiotic prophylaxis in maxillofacial trauma.	Prolonging antibiotic treatment over 24 h for surgically treated fractures does not appear to be beneficial. No evidence for antibiotic treatment in conservatively treated fractures.
Habib [18]	2019	SR/MA	13	Mandible Midface Orbital	high	low	low	high	high	RCTs and observational cohorts. Lack of age, onset and how reviewers were organized. Conclusion is mostly based on authors opinions and not their findings.	No support of routine use of postoperative antibiotic prophylaxis in patients with maxillofacial fractures.
Kyzas [33]	2011	SR	31	Mandible	high	high	high	high	high	Analyze 9 RCTs separately. Investigates systemic antibiotic prophylaxis in mandibular trauma.	Evidence to support the use of prophylactic antibiotics in mandible fractures is of poor quality.
Milic [3]	2021	SR	15	Mandible Maxilla Midface	high	high	high	high	high	Investigates antibiotic prophylaxis in oral and maxillofacial surgery.	Prophylactic antibiotic use is recommended in surgical extractions of third molars, comminute mandibular fractures, temporomandibular joint replacements, clean contaminated tumor removal, and complex implants but not in fractures of the upper or midface facial thirds.
Oppelaar [34]	2019	SR/MA	13	Mandible Maxilla Midface	low	low	low	low	low	Prospective. The only used search database was PubMed.	A short course of antibiotic treatment is recommended unless recorded ad-verse conditions.
Shridharani [26]	2015	SR	5	Mandible	unclear	low	unclear	high	unclear	Lack of aim, research question and PICO. No analysis of RoB.	Review demonstrates no postoperative antibiotic coverage in patients undergoing mandibular ORIF.

SR = systematic review; SR/MA = systematic review and Meta-analysis; ROBIS domain 1: aim and criteria for study selection; 2: identification and choice of studies; 3: assessment of studies and data extraction; 4: analyses and syntheses.

**Table 2:** Studies read in full text and assessed for eligibility but excluded from further analysis (*n* = 19).

Reference	Reason
Abubaker et al. [35]	Not systematic review
Andreasen et al. [36]	Not systematic review
Caruso et al. [37]	Not systematic review
Choi et al. [38]	Not systematic review
Cicuttin et al. [39]	Not systematic review
Doerr et al. [40]	Not systematic review
Gheza et al. [41]	Not systematic review
Grillo et al. [42]	Out of topic
Hermund et al. [43]	Out of topic
Kreutzer et al. [44]	Not systematic review
Legent et al. [45]	Not English
Martin et al. [46]	Not systematic review
Michel et al. [19]	Not systematic review
Meara et al. [47]	Not systematic review
Morris et al. [10]	Not systematic review
Mundinger et al. [25]	Not systematic review
Petersen et al. [48]	Not systematic review
Petersen et al. [49]	Not systematic review
Tucker et al. [50]	Out of topic

**Table 3:** Summary of results from studies with low risk of bias.

First Author	Year	Overall RoB	Total Sample Size (*n*)	Relative Risk of Infection (95% CI)			Conclusions According to Author
				Antibiotics vs no Antibiotics	1d vs >1d	Preop vs Pre- and Postop Antibiotics	
Dawoud [32]	2021	low	3285	1.38 (0.47–4.03)	0.84 (0.54–1.31)	1.47 (0.74–2.89)	- Seven of sixteen included studies were RCTs which were heterogenic and underpowered, *p* = 0.02, I^2^ = 69%. - No strong evidence for or against prophylactic antibiotic use in mandibular fractures.
Goormans [17]	2022	low	2430	N/A	N/A	N/A	- Prolonging antibiotic treatment over 24 h for surgically treated fractures does not appear to be beneficial (Infection Incidence Rate: 0 to 12.5%). No evidence for antibiotic treatment in conservatively treated fractures. - Well-constructed studies investigating type and duration of systemic antibiotics are lacking (High Risk of Bias: All included studies). - Results should be interpreted with caution because of limitations in all included studies.
Oppelaar [34]	2019	low	1974 (1133 on OMS treatment, 841 on ENT treatment.)	N/A	0.88 (95% CI, 0.63–1.21) to 0.89 (95% CI, 0.54–1.45)	N/A	- From a total of 21 included articles, 13 were analyzed regarding antibiotics in maxillofacial trauma, orthognathic surgery, wisdom-tooth surgery, and reconstructive surgery (RR, 0.88; 95%CI, 0.63–1.21). The remaining 8 analyzed skull base surgery, nose surgery and head and neck oncology surgery (RR, 0.89; 95%CI, 0.54–1.45). - More frequent adverse effects were found in the extended-course compared with the short-course antibiotic treatment (RR, 2.40; 95%CI, 1.20–3.54). - A short course of antibiotic treatment is recommended unless recorded adverse conditions.

N/A = Not available.

## Data Availability

Prospero 14/03/23 (https://www.crd.york.ac.uk/PROSPERO, accessed on 22 October 2025). Dnr: CRD42023404870.

## Supplementary Materials

The following supporting information can be downloaded at: https://www.mdpi.com/article/10.3390/cmtr18040048/s1, Table S1. Search strategy.

## Abbreviations

AMR	Antimicrobial resistance
ENT	Ear, Nose, and Throat
OMS	Oral and Maxillofacial Surgery
PICO	Participants, Intervention, Comparator, Outcome
PRISMA	Preferred Reporting Items for Systematic reviews and Meta Analyses
R	Review
RCT	Randomized Controlled Trial
ROBIS	Risk of Bias Analysis tool
SR	Systematic review
SR/MA	Systematic review and Meta analysis
TMJ	Temporomandibular joint
