# Supplementary material for: The Importance of Antibiotics in Facial Fracture Treatment—A Systematic Meta-Review
Source: Craniomaxillofac Trauma Reconstr. 2025 Nov 3;18(4):48. doi: 10.3390/cmtr18040048 (PMC12641708; doi:10.3390/cmtr18040048)
Supplement: Supplementary file 1 [file cmtr-18-00048-s001.zip › cmtr-3931172-supplementary.pdf]

Table S1. Search strategy**.**

	PubMed (Medline)	
1	(("Mandible"[Mesh] OR "Jaw"[Mesh] OR facial OR maxilla OR maxillofacial OR mandibular OR mandible OR maxillary OR craniomaxillofacial) AND ("Fractures, Bone"[Mesh] OR "Wounds and Injuries"[Mesh] OR fractures OR fracture OR fractured OR trauma OR injur*)) OR ("Facial Injuries"[Mesh])	121,817
2	(anti-bacterial agent[Title/Abstract] OR anti-bacterial agents[Title/Abstract] OR Antimycobacterial agents[Title/Abstract] OR Antimycobacterial agent[Title/Abstract] OR Bacteriocidal Agent[Title/Abstract] OR Bacteriocidal Agents[Title/Abstract] OR Anti-Bacterial Compounds[Title/Abstract] OR Anti-Bacterial Compound[Title/Abstract] OR antibiotics[Title/Abstract] OR antibiotic[Title/Abstract] OR microbial treatment[Title/Abstract] OR penicillin[Title/Abstract] OR amoxicillin[Title/Abstract] OR metronidazole[Title/Abstract] OR clindamycin[Title/Abstract] OR cephalosporin[Title/Abstract]) OR ("Anti-Bacterial Agents"[Mesh] OR "Cephalosporins"[Mesh] OR "Cephamycins"[Mesh] OR "Monobactams"[Mesh] OR "Carbapenems"[Mesh] OR "Thienamycins"[Mesh] OR "Penicillins"[Mesh] OR "Metronidazole"[Mesh] OR "Clindamycin"[Mesh] OR "Cephalosporins"[Mesh] OR "Anti-Infective Agents"[Mesh])	1,233,517
3	1 AND 2	3,894
4	systematic[ti] OR systematic review[pt] meta[ti] OR overview*[ti] OR review*[ti] OR review[pt]	3,923,811
5	3 AND 4	776
	Cochrane Library via Wiley	
1	((facial OR maxilla OR maxillofacial OR mandibular OR mandible OR maxillary OR craniomaxillofacial):ti,ab,kw OR MeSH descriptor: [Jaw] OR MeSH descriptor: [Mandible] explode all trees) AND (((fractures OR fracture OR fractured OR trauma OR injur*):ti,ab,kw OR MeSH descriptor: [Fractures, Bone] OR MeSH descriptor: [Wounds and Injuries] explode all trees)) OR MeSH descriptor: [Facial Injuries] explode all trees	3,634
2	(anti-bacterial agent OR anti-bacterial agents OR Antimycobacterial agents OR Antimycobacterial agent OR Bacteriocidal Agent OR Bacteriocidal Agents OR Anti-Bacterial Compounds OR Anti-Bacterial Compound OR antibiotics OR antibiotic OR microbial treatment OR penicillin OR amoxicillin OR metronidazole OR clindamycin OR cephalosporin):ti,ab,kw OR MeSH descriptor: [Anti-Bacterial Agents] OR MeSH descriptor: [Cephalosporins] OR MeSH descriptor: [Cephamycins] OR MeSH descriptor: [Monobactams] OR MeSH descriptor: [Carbapenems] OR MeSH descriptor: [Thienamycins] OR	82,014

Table S1. Search strategy.

	MeSH descriptor: [Penicillins] OR MeSH descriptor: [Metronidazole] OR MeSH descriptor: [Clindamycin] OR MeSH descriptor: [Cephalosporins] OR MeSH descriptor: [Anti-Infective Agents] explode all trees	
3	1 AND 2	234
	Cochrane reviews	10
	Web of Science via Clarivate	
1	TS=((facial OR maxilla OR maxillofacial OR mandibular OR mandible OR maxillary OR craniomaxillofacial) AND (facial OR maxilla OR maxillofacial OR mandibular OR mandible OR maxillary OR craniomaxillofacial))	399,032
	Intervention - antibiotics	
2	TS=(anti-bacterial agent OR anti-bacterial agents OR Antimycobacterial agents OR Antimycobacterial agent OR Bacteriocidal Agent OR Bacteriocidal Agents OR Anti-Bacterial Compounds OR Anti-Bacterial Compound OR antibiotics OR antibiotic OR microbial treatment OR penicillin OR amoxicillin OR metronidazole OR clindamycin OR cephalosporin OR Cephamycins OR Monobactams OR Carbapenems OR thienamycin OR Anti-Infective Agents OR Anti-Infective Agent)	759,204
3	1 AND 2	5,972
4	Publication Type: review article	703
